# Characterisation of a novel panel of polymorphic microsatellite loci for the liver fluke, *Fasciola hepatica*, using a next generation sequencing approach^☆^

**DOI:** 10.1016/j.meegid.2015.03.014

**Published:** 2015-06

**Authors:** Krystyna Cwiklinski, Katherine Allen, James LaCourse, Diana J. Williams, Steve Paterson, Jane E. Hodgkinson

**Affiliations:** a Veterinary Parasitology, Department of Infection Biology, Institute of Infection and Global Health, University of Liverpool, Liverpool L3 5RF, UK; b Liverpool School of Tropical Medicine, Pembroke Place, Liverpool L3 5QA, UK; c Institute of Integrative Biology, University of Liverpool, L69 7ZB, UK

**Keywords:** *Fasciola*, Microsatellite, Population structure, Next generation sequencing

## Abstract

The liver fluke, *Fasciola hepatica* is an economically important pathogen of sheep and cattle and has been described by the WHO as a re-emerging zoonosis. Control is heavily reliant on the use of drugs, particularly triclabendazole and as a result resistance has now emerged. The population structure of *F*. *hepatica* is not well known, yet it can impact on host–parasite interactions and parasite control with drugs, particularly regarding the spread of triclabendazole resistance. We have identified 2448 potential microsatellites from 83 Mb of *F*. *hepatica* genome sequence using msatfinder. Thirty-five loci were developed and optimised for microsatellite PCR, resulting in a panel of 15 polymorphic loci, with a range of three to 15 alleles. This panel was validated on genomic DNA from 46 adult *F*. *hepatica*; 38 liver flukes sourced from a Northwest abattoir, UK and 8 liver flukes from an established isolate (Shrewsbury; Ridgeway Research). Evidence for null alleles was found at four loci (Fh_1, Fh_8, Fh_13 and Fh_14), which showed markedly higher levels of homozygosity than the remaining 11 loci. Of the 38 liver flukes isolated from cattle livers (*n* = 10) at the abattoir, 37 genotypes were identified. Using a multiplex approach all 15 loci could be amplified from several life cycle stages that typically yield low amounts of DNA, including metacercariae, the infective life cycle stage present on pasture, highlighting the utility of this multiplex microsatellite panel. This study reports the largest panel of microsatellite markers available to date for population studies of *F*. *hepatica* and the first multiplex panel of microsatellite markers that can be used for several life cycle stages.

## Highlights

• 2448 microsatellite loci were identified within 83 Mb of *F*. *hepatica* genome sequence data.
• A panel of 15 polymorphic loci were developed and validated using genomic DNA from 46 parasites.
• The panel was developed and optimised as a multiplex PCR protocol.
• All loci could be amplified from several *F*. *hepatica* lifecycle stages with the multiplex approach.

## 1 Introduction

The liver fluke, *Fasciola hepatica*, is a zoonotic trematode parasite of grazing animals and humans. As the causative agent of fasciolosis, it results in significant global economic losses to the livestock industry and is considered a re-emerging human disease, currently included within the WHO top 17 listed neglected tropical diseases (Mas-Coma et al., 2009; WHO, 2006, 2007, 2012). Over the past decade the prevalence of *F*. *hepatica* infection has risen, in part due to climate change, increased animal movement and changing farming practices (van Dijk et al., 2010). Effective control and treatment of fasciolosis in both humans and livestock is reliant on anthelminitics, particularly the benzimidazole, triclabendazole (TCBZ); the drug of choice, given its superior efficacy against both the immature and mature parasites. However, reliance on TCBZ has led to widespread resistance (Overend and Bowen, 1995; Fairweather, 2005; Brennan et al., 2007).

*Fasciola* has a complex life cycle that involves an intermediate host, the mud snail *Galba truncatula* and multiple definitive hosts (Supplementary Fig. 1). The ability of *F*. *hepatica* to infect a number of different mammalian hosts is considered to be one of the factors responsible for its extensive geographical reach (Mas-Coma, 2004; Mas-Coma et al., 2005; Robinson and Dalton, 2009; Furst et al., 2012). Eggs are passed onto the pasture in faeces from the definitive host. Following embryonation, miracidia hatch from the eggs and infect the snail intermediate host, where a clonal expansion of the parasite occurs, through sporocysts, rediae and cercariae life cycle stages. Following release from the snail the infective stage, the metacercariae, encyst on herbage prior to being ingested by the definitive host. Juvenile flukes migrate to the bile ducts, where they reach sexual maturity. In the mammalian host, the adult parasites undergo a complex reproductive strategy, with the potential for self and cross fertilisation and parthenogenesis.

The asexual multiplication of *F*. *hepatica* within the snail host and complex reproductive biology in the mammalian host facilitates high gene flow and has the potential to promote high levels of genetic variability within *F*. *hepatica* populations. Alternatively, multiple permissive hosts may lead to host selection of *F*. *hepatica* populations resulting in population sub-structuring and/or differential genetic diversity in different hosts and geographical locations. How population structure may affect the spread of TCBZ resistance throughout a population has yet to be investigated. To fully understand the impact and spread of TCBZ resistance or any genetic component that provides a survival advantage, the population structure of *F*. *hepatica* needs to be investigated, using a large panel of polymorphic markers that are neutrally inherited. This information is needed to determine how population dynamics impact on host-parasite interactions and on parasite control interventions.

Studies of the genetic structure of closely related trematodes, including *Clonorchis sinensis*, *Opisthorchis viverrini* and *Schistosoma* spp. have relied on a variety of molecular tools, in particular panels of microsatellite markers, to elucidate population genetic structure (Gower et al., 2007, 2011; Agola et al., 2009; Valentim et al., 2009; Laoprom et al., 2010; Xiao et al., 2011; Glenn et al., 2013; Steinauer et al., 2010). The majority of studies investigating the genetic structure of *F*. *hepatica* populations involve the application of mitochondrial markers and/or small panels of microsatellites (reviewed by Hodgkinson et al., 2013). To date only five polymorphic microsatellites have been developed and applied to *Fasciola* populations in Spain and Eqypt (Hurtrez-Boussès et al., 2004; Dar et al., 2011, 2013; Vilas et al., 2012). With the advancement of next generation technologies, trematode genome datasets are now being mined for microsatellite markers to develop panels of polymorphic markers; for example *Schistosoma japonicum* (Xiao et al., 2011) and more recently *Fascioloides magna* (Minarik et al., 2014). Here, we demonstrate how 83 Mb of 454 FLX *F*. *hepatica* genome sequence data was used to generate a resource of >2000 potential microsatellites, with suitable flanking regions for primer design. Thirty-five loci were selected for preliminary screening, resulting in the development and characterisation of a panel of 15 polymorphic markers. Genomic organisation of the panel of 15 microsatellite loci was investigated by mining our recently generated 1.3 Gb *F*. *hepatica* draft genome. Importantly, following an initial validation of these markers their utility was demonstrated for multiple *F*. *hepatica* life cycle stages that typically yield low amounts of DNA, particularly eggs, miracidia and metacercariae stages; greatly facilitating population-level analyses across multiple geographical locations in future.

## 2 Methods

### 2.1 *F*. *hepatica* isolates

For development and validation of the microsatellite panel, 46 adult *F*. *hepatica* parasites were obtained from two sources: (1) 38 adult liver flukes were recovered from livers of 10 naturally infected cattle at an abattoir, in Northwest England, ranging between one and seven adult liver flukes from each liver; (2) eight adult liver flukes were tested from two cattle experimentally infected with the Shrewsbury isolate (Ridgeway Research), with one and seven adult liver flukes from each animal, respectively. Each adult liver fluke was genotyped individually. Eggs were harvested from adult *F*. *hepatica* parasites obtained from experimentally infected animals, following incubation in 1–2 ml of Dulbecco’s Modified Eagle’s Media (DMEM; Sigma Aldrich) for a minimum of 2 h at 37 °C. A subset of eggs was embryonated by incubation at 26 °C for 12 days. Following embryonation the eggs were hatched to release miracidia using standard *F*. *hepatica* protocols. Metacercariae were shed from experimentally infected colonies of *G*. *truncatula* maintained at the University of Liverpool.

### 2.2 DNA extraction and quantification

Genomic DNA extraction was carried out using the DNeasy Blood and Tissue Kit (Qiagen) for eggs, miracidia, metacercariae and adults or Genomic tip 100G kit (Qiagen; adult parasites only), according to the manufacturer’s instructions. Fifty eggs/miracidia/metacerariae were used for each extraction and DNA was eluted into 60 μl of AE buffer (DNeasy Blood and Tissue Kit; Qiagen). Whole adult liver flukes or a 20 mg section of adult liver fluke near the anterior sucker was used for extraction, followed by an ethanol precipitation and re-suspension of the DNA in 60 μl TE (pH 8.0). Chelex DNA extractions on single miracidia were carried out according to Valentim et al. (2009), followed by whole genome amplification using the illustra GenomiPhi V2 DNA Amplification Kit in a reaction volume of 50 μl, according to the manufacturer’s instructions (GE Healthcare Life Sciences). All DNA concentrations were calculated using the Quant-iT™ PicoGreen® dsDNA assay kit (Life Technologies).

### 2.3 Genome sequencing and microsatellite identification

*F*. *hepatica* genome sequence data was generated from genomic DNA extracted from a single adult parasite (Sligo isolate; DNeasy Blood and Tissue Kit, Qiagen), using 454 FLX sequencing technology (Roche; Centre for Genomic Research, University of Liverpool). The resulting 450 Mb of raw data was assembled into 190,089 contigs, representing 83 Mb of sequence data (ENA accession number PRJEB8349), which was mined for microsatellites using msatfinder (www.genomics.ceh.ac.uk/msatfinder; default thresholds; Thurston and Field, 2005). More than 2000 potential microsatellites were identified comprising of di- to hexa-nucleotide repeats with suitable flanking regions for primer design (Supplementary Table 1). The tri-and tetra- nucleotide motifs were selected for further analysis, as they are known to be less prone to enzyme slippage during PCR, which can make allele designation difficult (Edwards et al., 1991; Guichoux et al., 2011). Compound microsatellites were excluded and the remaining microsatellites were sorted according to the number of tandem repeats as per Xiao et al. (2011). Thirty-five loci were selected for preliminary PCR screening, which resulted in 15 loci being taken forward for PCR amplification using a modified semi-nested PCR protocol based on Schuelke’s method (Schuelke, 2000). Genomic organisation of the panel of 15 microsatellite loci was investigated by mining the recently generated 1.3 Gb *F*. *hepatica* draft genome sequenced from the Shrewsbury isolate (ENA accession numbers LN627018-LN647175: assembly data and PRJEB6687: genomic read data; Cwiklinski et al., in press), using BLAST (NCBI) and manual curation.

### 2.4 Microsatellite PCR and capillary sequencing

Primary and secondary amplification from adult genomic DNA was carried out in a 10 μl reaction volume, containing 5 μl of 2× BioMix Red master mix (Bioline). The primary PCR contained 1 μl of adult DNA template (∼10 ng) and 0.5 μM of both the forward M13-tagged primer and the reverse primer (Table 1; Eurofins MWG Operon). The cycling conditions were an initial 10 min at 94 °C, followed by 25 cycles (see Table 1) at 94 °C for 30 s, annealing temperature (see Table 1) for 45 s and 72 °C for 45 s, with a final extension at 72 °C for 10 min. The secondary PCR contained 1 μl of the primary PCR, 1 μM of the fluorescent dye-M13 tagged primer (5′ 6-FAM/NED/PET/VIC - TGTAAAACGACGGCCAGT 3′; Life Technologies) and 0.5 μM of the reverse primer (the same used in the primary PCR). The cycling conditions were as above for 10 cycles with an annealing temperature of 53 °C. To take advantage of multiplex PCR protocols that offer distinct advantages when low quantities of DNA are available, the 15 microsatellite markers were subsequently validated using a multiplex PCR approach. Four multiplex reactions were optimised using the Type-it Microsatellite PCR kit (Qiagen) according to the manufacturer’s instructions (30 cycles; Supplementary Table 2), using 1 μl of adult genome DNA template. Microsatellite amplification for the egg, miracidia and metacercariae life cycle stages was carried out using 3 μl of the DNA template; with an increased number of PCR cycles for both the semi-nested PCR (see Table 1) and multiplex PCR approaches (40 cycles; Supplementary Table 2).

PCR products were analysed by agarose gel electrophoresis (2% gel) using SYBR® Safe DNA stain (Life Technologies). If a positive result was obtained by gel electrophoresis, the PCR products were diluted 50-fold and 1 μl of this dilution multiplexed in Hi-Di Formamide (8.8 μl; Life Technologies) with GeneScan LIZ500 size standards (0.2 μl; Life Technologies), prior to sequencing using a 3100 Genetic Analyzer capillary electrophoresis system (Life Technologies).

### 2.5 Data analysis

Sequence size was determined with an ABI PRISM 3100 DNA analyzer and Peak Scanner v1.0 software. Analysis was carried out using GENEPOP version 4.0.10 (Raymond and Rousset, 1995; Rousset, 2008), Genetix (Belkhir et al., 2004) and Micro-Checker (van Oosterhout et al., 2004). Each of the markers produced clear, unambiguous traces for individual parasites as expected for single locus markers in a diploid organism.

## 3 Results

### 3.1 Validation of *F*. *hepatica* microsatellite panel

The 83 Mb *F*. *hepatica* genome sequence data, comprised of 190,089 contigs was mined for microsatellite markers resulting in 5222 potential microsatellite loci; 2448 of which were identified with suitable primer flanking regions. To investigate the utility of these microsatellites as a tool for population level studies a subset of 15 loci were validated using 46 individual adult parasites from cattle; 38 liver flukes sourced from naturally infected cattle and eight liver flukes sourced from experimentally infected cattle. All 15 loci studied were found to be polymorphic, with allele numbers ranging from three-15 (Table 2; Supplementary Table 3). For the 38 liver flukes isolated from naturally infected cattle, 37 multi-locus genotypes (MLG) were reported. Hardy–Weinberg equilibrium (HWE) and the heterozygote deficiency for each locus were examined using the exact test, using GENEPOP version 4.0.10 (Table 2). Three loci (Fh_1, Fh_8 and Fh_14) were found to significantly deviate from HWE, following Bonferroni’s adjustment (*P* < 0.00005; Rice, 1989). All were significant for heterozygote deficiency (*P* < 0.00005). In addition four loci (Fh_1, Fh_8, Fh_13 and Fh_14) were found to have potential null alleles (as observed by plots of *F*_IS_ vs number of alleles and *F*_IS_ vs heterozygosity (Supplementary Fig. 2). These loci together within an additional two loci (Fh_4 and Fh_11) were also identified as potential null alleles (calculated using Micro-Checker). All 15 loci were consistently amplified, produced unambiguous traces and were sufficiently polymorphic to provide a robust panel of microsatellites for accurately genotyping *F*. *hepatica* parasites.

### 3.2 Genotyping multiple *F*. *hepatica* life cycle stages

To fully exploit their potential the microsatellite markers were validated as a means of genotyping several life cycle stages of *F*. *hepatica*; namely eggs, miracidia and metacercariae. A subset of eight loci consistently and accurately genotyped pools of 50 metacercariae (Fh_1, Fh_2, Fh_3, Fh_4, Fh_7, Fh_12, Fh_13, Fh_14 and Fh_15; Table 1) and three loci in particular (Fh_1, Fh_2, Fh_3) could reliably genotype fewer than 50 metacercariae. Following this initial validation the panel was tested on duplicate samples of eggs, miracidia and metacercariae (50 of each) from four different isolates, whose genetic profile was known. Microsatellite analysis of these samples showed that the resulting genotype was consistent with each isolate profile. Multiple alleles for each locus were represented within each population of eggs indicating the markers were capable of defining populations of parasites. To improve microsatellite amplification from these life cycle stages that typically yield low levels of DNA, a multiplex protocol was used, which resulted in consistent amplification for the whole panel of 15 loci. Finally DNA extraction and subsequent amplification of microsatellites was optimised for a single miracidium, using Chelex DNA extraction with or without subsequent whole genome amplification. DNA was extracted from 10 individual replicate miracidia of known genotype and the expected profile was consistently amplified either directly or following whole genome amplification, suggesting that individual miracidia can be successfully genotyped using this approach.

### 3.3 Genomic location of *F*. *hepatica* microsatellites

The microsatellites were originally identified in our preliminary 83 Mb *F*. *hepatica* genome dataset and their presence and location was subsequently confirmed by mining our recently generated 1.3 Gb *F*. *hepatica* draft genome (Cwiklinski et al., in press). All loci were detected and found to reside on discrete scaffolds that ranged in size from 33 to 830 Mbp indicating that these loci are dispersed throughout the genome (Table 3). Linkage disequilibrium (LD) analysis carried out on the subset of nine loci where potential null alleles were not observed according to the more conservative Micro-Checker analysis (Fh_2, Fh_3, Fh_5, Fh_6, Fh_7, Fh_9, Fh_10, Fh_12, and Fh_15), showed significant results for several microsatellite pairs (Table 4).

## 4 Discussion

This study reports the characterisation of a panel of 15 novel polymorphic microsatellites, constituting the largest panel of microsatellite markers available for *F*. *hepatica*. These loci were identified from within a database of >2000 microsatellite loci, which was compiled from 83 Mb *F*. *hepatica* genome sequence data. Following the sequencing of the complete *F*. *hepatica* genome (Cwiklinski et al., in press), we now know that 83 Mb of sequence data represents only ∼6.5% of the 1.3 Gb draft *F*. *hepatica* genome. The identification of this large number of microsatellite loci from such a small proportion of genomic sequence shows the potential of next generation sequencing resources to facilitate population genetic studies in the future.

The liver fluke samples chosen for the validation of these loci were sourced from 12 different animals, 10 of which were naturally infected with *F*. *hepatica*. All of the parasites obtained from each of the 10 naturally infected animals were used for this study, a total of 38 liver flukes, to allow an initial observation of the genetic profile of liver flukes found in an animal. Eight liver flukes from two experimentally infected animals were also tested, which was consistent with the number of adult liver flukes observed in a naturally infected cow.

Statistical analysis showed that these loci are polymorphic with a wide range of alleles, highlighting the suitability of these loci to further understand the population genetics of *F*. *hepatica*. Null alleles were potentially identified for at least four loci (Fh_1, Fh_8, Fh_13 and Fh_14), which exhibited notably higher levels of homozygosity (measured by *F*_IS_) than the remaining 11 loci. Whereas parthenogenesis in *Fasciola* is known and could lead to higher inbreeding and *F*_IS_ across the genome, the higher levels of *F*_IS_ observed during this study at the subset of four loci suggests locus-specific effects, for which the presence of null alleles is a parsimonious explanation. A further two loci were potentially identified using the Micro-Checker algorithm (Fh_4 and Fh_11). Given the complex nature of the *Fasciola* life cycle the loci that have been identified as null alleles would need to be verified in a larger study and any future statistical analysis interpreted with caution, especially if the method of reproduction (asexual vs sexual) is not known for the specific isolate. It should be noted that analysis of the UK isolates used for this particular study have been shown to reproduce sexually and are diploid (Beesley et al., unpublished).

Several loci pairs were found to have significant LD values. As these values could be influenced by the population structure within this panel, specifically by those loci that were not in HWE, those loci showing potential null alleles were omitted from this analysis. The fact that the samples used for this analysis came from several animals and therefore from several potential populations of *F*. *hepatica*, could also explain the LD values. LD was not calculated per animal due to the small number of liver flukes obtained. Analysis of a larger population would give a more accurate representation of the LD. Analysis of the location of the 15 loci within the draft *F*. *hepatica* genome has shown that they are present on discrete scaffolds. Although the loci are thought not to be closely located, those loci with significant LD values could be present in areas of the genome that are co-inherited. Also as the genome is still in a draft format and with a wide range of scaffold sizes (33–830 Mbp), these loci may be more closely located than presently thought. Further annotation of the scaffolds where the loci are located is required to investigate the genes closely located to the microsatellite loci on the scaffolds and how this may affect their heritability. With the availability of single molecule sequencing, the assembly of the *F*. *hepatica* genome can also be improved, providing further information regarding the location of these loci.

An important aspect of this study involved the optimisation of the microsatellite panel for three different life cycle stages; eggs, miracidia and metacercariae. Typically these stages yield low amounts of DNA and are a significant challenge to investigate, yet are important as they represent the more readily accessible stages of the parasite on pasture, avoiding the need to passage cercariae through a mammalian host (Supplementary Fig. 1). Existing studies have concentrated on the adult stage of the *F*. *hepatica* life cycle but the ability of our microsatellite panel to robustly genotype multiple life cycle stages, potentially at the single miracidium level, offers significant promise for population genetic, molecular epidemiology and studies of the transmission dynamics of *F*. *hepatica* in future. Eight loci were shown to be robust, being consistently amplified from samples of 50 eggs/miracidia/metacercariae. In fact prior to multiplexing the markers, three of the loci could be amplified from fewer than 50 metacercariae (Fh_1, Fh_2 and Fh_3). It should be noted that although using pools of parasite material (specifically eggs and miracidia from populations of *F*. *hepatica*) allows the extraction of sufficient DNA for microsatellite analysis, this method can only be used to give an overall representation of the potential alleles present within those populations. Capillary sequencing of microsatellite PCRs can often result in stutter peaks, n-1 peaks or large allelic drop-out, which could result in a greater number of alleles for pooled samples from a given population, though the multiplex protocol was optimised to minimise these effects. Microsatellite analysis of individual miracidia or metacercariae resulting from single snail: miracidia infections, although more time consuming, would give a more accurate representation of the number of alleles present within a population. With this in mind we adopted the protocol of Valentim et al. (2009), that involves extracting DNA using Chelex followed by whole genome amplification, to successfully genotype DNA from individual *F*. *hepatica* miracidia using our panel of 15 markers. These results show the potential power of this method for *F*. *hepatica* populations, although further analysis is required using greater numbers of miracidia from different populations to accurately determine the error rate using this method for natural populations.

Levels of genetic variability within populations of the *Fasciola* genus from different hosts and geographical locations are currently not well understood, in particular, the impact of population dynamics on the development of anthelmintic resistance. To date the majority of studies investigating the genetic structure of *Fasciola* populations have focussed on available mitochondrial markers. Sequencing of the complete *F*. *hepatica* mitochondrial genome of two geographically distinct isolates (Australia and Salt Lake City) showed that these genomes demonstrate levels of intra-specific variation <1% of the 14 kb mitochondrial genome (Le et al., 2001). Mitochondrial markers have been applied to investigate TCBZ resistance in cattle in the Netherlands (Walker et al., 2011) and Teofanova and colleagues have shown that partial mitochondrial DNA sequences are also useful for population genetics analysis (Teofanova et al., 2011). However, it should be noted that mitochondrial markers have been shown to have potential limitations, including the fact that direct and indirect selection can influence mitochondria resulting in mitochondrial DNA not always being a strict neutral marker (Ballard and Whitlock, 2004; Galtier et al., 2009). Coupled with the fact that *F*. *hepatica* has a complex life cycle, including an asexual component all *Fasciola* spp. population genetics data should be interpreted with caution.

Prior to this study only a small panel of microsatellites was available (Hurtrez-Boussès et al., 2004). Several of these loci were tested as part of this study, with inconsistent results (data not shown). This original panel was developed using *F*. *hepatica* parasites from the Bolivian Altiplano. These results could indicate that there is potential polymorphism at the microsatellite primer sites between geographically distinct *Fasciola* spp. isolates, and so the panel outlined within this study should be tested on samples from different geographical areas from large populations to give an accurate representation of the utility of this panel and the population genetic structure of *F*. *hepatica* isolates.

Studies have shown that microsatellites developed for one species can often be used in related species (Jarne and Lagoda, 1996; Gemmell et al., 1997; Dar et al., 2011). In particular one study showed that the *F*. *hepatica* loci identified by Hurtrez-Boussès et al. (2004) could be used for the related species *Fasciola gigantica* (Dar et al., 2011), indicating the potential of the microsatellite panel described within this study to be used for other *Fasciola* species. Nevertheless, with the expansion of next generation sequencing technologies, large genomic datasets are more readily available for mining, particularly for genetic markers, providing potential for a *F*. *gigantica*-specific microsatellite panel.

The availability of this polymorphic panel will allow extensive study of the population structure of *F*. *hepatica*. In particular these markers will provide insight into geographically distinct isolates, advancing our understanding of a parasite which has a complex life cycle and the ability to infect a number of mammalian hosts. The availability of this panel of microsatellite loci also allows the population structure of *F*. *hepatica*, found in areas of differential exposure to TCBZ, to be explored, which will be invaluable for the continued study of TCBZ resistance.

## Figures and Tables

**Table 1:** Primer sequences and properties of *F*. *hepatica* (Fh) microsatellite alleles for the modified Schuelke’s method.

Locus	Primer sequence (5′–3′)	Dye	*T*_a_ (°C)	No. PCR cycles	
				Adult DNA template	Other DNA templates^
Fh_1⁎	F: 5′-TGTAAAACGACGGCCAGTCCCATGGTGTTGCACAGAT-3′ R: 5′-CTTCACCAAAAGCCGCTAAC-3′	VIC	55	25	45
Fh_2⁎	F: 5′-TGTAAAACGACGGCCAGTTGAGAAACTGATTCACCGACTG-3′ R: 5′-GAGCTTGTGCTCTCGGAACTA-3′	VIC	57	25	45
Fh_3⁎	F: 5′-TGTAAAACGACGGCCAGTCACGGCAACTGATGAATGAA-3′ R: 5′-TCCTCGTTTTTGGACCTCAG-3′	PET	58	25	45
Fh_4	F: 5′-TGTAAAACGACGGCCAGTTCACGAAATTGGAGACGACA-3′ R: 5′-TGCATGCAAGAATTGTACCC-3′	PET	59	25	/
Fh_5	F: 5′-TGTAAAACGACGGCCAGTCATCACCACTGTCTTCGATCA-3′ R: 5′-CGAAGCATTGATAAGATTTCCA-3′	6-FAM	57	25	/
Fh_6	F: 5′-TGTAAAACGACGGCCAGTACGTCCGTCCGTTAAGTGAG-3′ R: 5′-TTTGAGGTCGACATCCTTCA-3′	6-FAM	55	25	/
Fh_7⁎	F: 5′-TGTAAAACGACGGCCAGTTGCACTCTAGCATGGTTTGG-3′ R: 5′-AAGTCTTCAGTGCCCCTTCC-3′	6-FAM	57	25	45
Fh_8	F: 5′-TGTAAAACGACGGCCAGTCTCCTGAGGATGATCGGAAA-3′ R: 5′-CTACCGGATCGTTTTGACCA-3′	NED	57	25	/
Fh-9	F: 5′-TGTAAAACGACGGCCAGTAACCCGTATCACCACCAAAC-3′ R: 5′-CTCCCAATCCTGCCATACAT-3′	PET	58	25	/
Fh_10	F: 5′-TGTAAAACGACGGCCAGTTTTAGTCGCGGAGCTACCAT-3′ R: 5′-CCACTTTCGTCATGCACATT-3′	NED	57	25	/
Fh_11	F: 5′-TGTAAAACGACGGCCAGTTAAACCGTTGCTTCACGTTG-3′ R: 5′-CAAAGTGTTTGGCGAGCTG-3′	PET	57	25	/
Fh_12⁎	F: 5′-TGTAAAACGACGGCCAGTCCACGAGAAGTGGAATTCGT-3′ R: 5′-GTAGGTCCACTCCCTGTCCA-3′	VIC	60	25	50
Fh_13⁎	F: 5′-TGTAAAACGACGGCCAGTGAAACTGTCCCGAAAACGAG-3′ R: 5′-GCGTGCAACATAGGTGAAAA-3′	PET	55	25	50
Fh_14⁎	F: 5′-TGTAAAACGACGGCCAGTACAGGGCTTTGAAGCATGAC-3′ R: 5′-GGGATAGAGCCGTACTGGAA-3′	NED	57	25	50
Fh_15⁎	F: 5′-TGTAAAACGACGGCCAGTAATGCGGAAAAGAGCGATTA-3′ R: 5′-GAAATTGGGAGCAACTGCAT-3′	NED	55	25	55

Primer sequence: sequence underlined represents the M13 sequence tag. *T*_a_: annealing temperature for PCR.

⁎ Loci that can be consistently amplified from egg, miracida and metacercariae DNA.

^ DNA extracted from egg, miracida and metacercariae life cycle stages.

**Table 2:** Statistical analysis of the 15 *F*. *hepatica* microsatellite loci.

Locus	Repeat motif	Locus size (bp)	No. of alleles	Allele size range (bp)	*H*_E_	*H*_O_	*F*_IS_	HW
Fh_1	TTTG	201	4	200–224	0.564	0.196	**0.656**	**0.000**
Fh_2	TTGA	213	16	200–356	0.826	0.733	0.087	0.134
Fh_3	AAAC	196	5	195–219	0.655	0.651	−0.030	0.088
Fh_4	TAA	186	13	178–214	0.859	0.739	0.140	0.002
Fh_5	ACT	159	15	166–338	0.850	0.800	0.057	0.071
Fh_6	TAT	180	15	193–265	0.861	0.913	−0.061	0.486
Fh_7	TAT	177	13	178–259	0.759	0.698	0.081	0.593
Fh_8	ATA	167	11	180–213	0.821	0.533	**0.353**	**0.000**
Fh-9	GTT	203	3	210–225	0.332	0.400	−0.117	0.032
Fh_10	TAA	217	12	215–251	0.790	0.891	−0.130	0.210
Fh_11	ATA	211	14	209–254	0.877	0.711	0.191	0.000⁎
Fh_12	ATC	215	8	219–258	0.705	0.652	0.075	0.921
Fh_13	CAT	192	4	202–226	0.648	0.444	0.327	0.002
Fh_14	AAT	201	10	202–238	0.808	0.318	**0.634**	**0.000**
Fh_15	TATG	206	7	213–277	0.598	0.630	−0.071	0.002

Locus size: represents the size of the locus found in the *F*. *hepatica* genomic sequence data using msatfinder. *H*_E_: expected heterozygosity and *H*_O_: observed heterozygosity; calculated using GENETIX. *F*_IS_: inbreeding coefficient, according to Weir and Cockerham (1984) and HW: Fisher’s exact test for Hardy–Weinberg equilibrium, *P* < 0.00005 after Bonferroni’s adjustment; calculated using GENEPOP. In bold are the three loci that significantly deviate from HWE following Bonferroni's adjustment (*P* < 0.00005).

⁎ Not significant after Bonferroni’s adjustment (0.0002).

**Table 3:** Location of microsatellite loci within the draft *F*. *hepatica* genome⁎.

Loci	Location in draft genome (ENA scaffold number)	Size of scaffold/bp
Fh_1	LN629046	119,809
Fh_2	LN627942	830,113
Fh_3	LN627685	203,809
Fh_4	LN627551	495,632
Fh_5	LN627876	548,152
Fh_6	LN629193	178,014
Fh_7	LN635535	32,950
Fh_8	LN629482	110,497
Fh_9	LN628826	266,251
Fh_10	LN628015	227,781
Fh_11	LN629076	121,942
Fh_12	LN628360	220,783
Fh_13	LN630045	119,190
Fh_14	LN628821	190,238
Fh_15	LN627509	452,066

⁎ Cwiklinski et al., in press. Data are freely available from the European Nucleotide Archive under accessions LN627018-LN647175 (assembly data), PRJEB6687 (genomic read data).

**Table 4:** Genotypic linkage disequilibrium for the nine *F*. *hepatica* microsatellite loci where null alleles were not identified.

Fh pairs	*P*-value⁎
Fh_2 & Fh_6	0.000000
Fh_2 & Fh_7	0.000000
Fh_2 & Fh_10	0.000054
Fh_2 & Fh_12	0.000000
Fh_3 & Fh_6	0.000000
Fh_3 & Fh_10	0.000005
Fh_3 & Fh_12	0.001212
Fh_5 & Fh_6	0.000118
Fh_5 & Fh_7	0.000000
Fh_5 & Fh_10	0.000724
Fh_5 & Fh_12	0.001220
Fh_6 & Fh_10	0.000000
Fh_6 & Fh_12	0.000000
Fh_10 & Fh_12	0.000095

⁎ *P* < 0.05 following Bonferroni’s adjustment = *P* < 0.0014238.

## References

[b0005] Agola L.E., Steinauer M.L., Mburu D.N., Mungai B.N., Mwangi I.N., Magoma G.N., Loker E.S., Mkoji G.M. (2009). Genetic diversity and population structure of *Schistosoma mansoni* within human infrapopulations in Mwea, central Kenya assessed by microsatellite markers. Acta Trop..

[b0010] Ballard J.W., Whitlock M.C. (2004). The incomplete natural history of mitochondria. Mol. Ecol..

[b0225] Belkhir K., Borsa P., Chikhi L., Raufaste N., Bonhomme F. (2004). GENETIX 4.05, Logiciel Sous Windows TM Pour La Génétique Des Populations. Laboratoire Génome, Populations, Interactions.

[b0020] Brennan G.P., Fairweather I., Trudgett A., Hoey E., McCoy, McConville M., Meaney M., Robinson M., McFerran N., Ryan L., Lanusse C., Mottier L., Alvarez L., Solana H., Virkel G., Brophy P.M. (2007). Understanding triclabendazole resistance. Exp. Mol. Pathol..

[b9000] Cwiklinski, K., Dalton, J.P., Dufresne, P.J., La Course, J., Williams, D., Hodgkinson, J.E., Paterson S., in press. The Fasciola hepatica genome: gene duplication and polymorphism reveals adaptation to the host environment and capacity for rapid evolution, Genome Biol. 10.1186/s13059-015-0632-2 PMC4404566 25887684

[b0025] Dar Y., Amer S., Courtioux B., Dreyfuss G. (2011). Microsatellite analysis of *Fasciola* spp. in Egypt. Parasitol. Res..

[b0030] Dar Y., Lounnas M., Djuikwo Teukeng F.F., Mouzet R., Courtioux B., Hurtrez-Bousses S., Vignoles P., Dreyfuss G., Rondelaud D. (2013). Variations in local adaptation of allopatric *Fasciola hepatica* to French *Galba truncatula* in relation to parasite origin. Parasitol. Res..

[b0035] Edwards A., Civitello A., Hammond H.A., Caskey C.T. (1991). DNA typing and genetic mapping with trimeric and tetrameric tandem repeats. Am. J. Hum. Genet..

[b0040] Fairweather I. (2005). Triclabendazole: new skills to unravel an old(ish) enigma. J. Helminthol..

[b0045] Furst T., Duthaler U., Sripa B., Utzinger J., Keiser J. (2012). Trematode infections: liver and lung flukes. Infect. Dis. Clin. North Am..

[b0050] Galtier N., Nabholz B., Glemin S., Hurst G.D. (2009). Mitochondrial DNA as a marker of molecular diversity: a reappraisal. Mol. Ecol..

[b0055] Gemmell N.J., Allen P.J., Goodman S.J., Reed J.Z. (1997). Interspecific microsatellite markers for the study of pinniped populations. Mol. Ecol..

[b0060] Glenn T.C., Lance S.L., McKee A.M., Webster B.L., Emery A.M., Zerlotini A., Oliveira G., Rollinson D., Faircloth B.C. (2013). Significant variance in genetic diversity among populations of *Schistosoma haematobium* detected using microsatellite DNA loci from a genome-wide database. Parasit. Vectors.

[b0065] Gower C.M., Gabrielli A.F., Sacko M., Dembele R., Golan R., Emery A.M., Rollinson D., Webster J.P. (2011). Population genetics of *Schistosoma haematobium*: development of novel microsatellite markers and their application to schistosomiasis control in Mali. Parasitology.

[b0070] Gower C.M., Shrivastava J., Lamberton P.H., Rollinson D., Webster B.L., Emery A., Kabatereine N.B., Webster J.P. (2007). Development and application of an ethically and epidemiologically advantageous assay for the multi-locus microsatellite analysis of *Schistosoma mansoni*. Parasitology.

[b0075] Guichoux E., Lagache L., Wagner S., Chaumeil P., Leger P., Lepais O., Lepoittevin C., Malausa T., Revardel E., Salin F., Petit R.J. (2011). Current trends in microsatellite genotyping. Mol. Ecol. Resour..

[b0080] Hodgkinson J., Cwiklinski K., Beesley N.J., Paterson S., Williams D.J. (2013). Identification of putative markers of triclabendazole resistance by a genome-wide analysis of genetically recombinant *Fasciola hepatica*. Parasitology.

[b0085] Hurtrez-Boussès S., Durand P., Jabbour-Zahab R., Guégan J., Meunier C., Bargues M., Mas-Coma S., Renaud F. (2004). Isolation and characterization of microsatellite markers in the liver fluke (*Fasciola hepatica*). Mol. Ecol. Notes.

[b0090] Jarne P., Lagoda P.J. (1996). Microsatellites, from molecules to populations and back. Trends Ecol. Evol..

[b0095] Laoprom N., Sithithaworn P., Ando K., Sithithaworn J., Wongkham S., Laha T., Klinbunga S., Webster J.P., Andrews R.H. (2010). Microsatellite loci in the carcinogenic liver fluke, *Opisthorchis viverrini* and their application as population genetic markers. Infect. Genet. Evol..

[b0100] Le T.H., Blair D., McManus D.P. (2001). Complete DNA sequence and gene organization of the mitochondrial genome of the liverfluke, *Fasciola hepatica* L. (Platyhelminthes; Trematoda). Parasitology.

[b0220] Mas-Coma S., Cotruvo J.A., Dufour A., Rees G., Bartram J., Carr R., Cliver D.O., Craun G.F., Fayer R., Gannon V.P.J. (2004). Human fasciolosis. Waterborne Zoonoses: Identification, Causes and Control.

[b0115] Mas-Coma S., Bargues M.D., Valero M.A. (2005). Fascioliasis and other plant-borne trematode zoonoses. Int. J. Parasitol..

[b0120] Mas-Coma S., Valero M.A., Bargues M.D. (2009). Chapter 2. Fasciola, lymnaeids and human fascioliasis, with a global overview on disease transmission, epidemiology, evolutionary genetics, molecular epidemiology and control. Adv. Parasitol..

[b0125] Minarik G., Bazsalovicsova E., Zvijakova L., Stefka J., Palkova L., Kralova-Hromadova I. (2014). Development and characterization of multiplex panels of polymorphic microsatellite loci in giant liver fluke *Fascioloides magna* (Trematoda: Fasciolidae), using next-generation sequencing approach. Mol. Biochem. Parasitol..

[b0130] Overend D.J., Bowen F.L. (1995). Resistance of *Fasciola hepatica* to triclabendazole. Aust. Vet. J..

[b0135] Raymond M., Rousset F. (1995). GENEPOP (version 1.2): population genetics software for exact tests and ecumenicism. J. Heredity.

[b0140] Rice W.R. (1989). Analysing tables of statistical tests. Evolution.

[b0145] Robinson M.W., Dalton J.P. (2009). Zoonotic helminth infections with particular emphasis on fasciolosis and other trematodiases. Philos. Trans. R. Soc. Lond. B Biol. Sci..

[b0150] Rousset F. (2008). Genepop’007: a complete re-implementation of the genepop software for Windows and Linux. Mol. Ecol. Resour..

[b0155] Schuelke M. (2000). An economic method for the fluorescent labeling of PCR fragments. Nat. Biotechnol..

[b0160] Steinauer M.L., Blouin M.S., Criscione C.D. (2010). Applying evolutionary genetics to schistosome epidemiology. Infect. Genet. Evol..

[b0165] Teofanova D., Kantzoura V., Walker S., Radoslavov G., Hristov P., Theodoropoulous G., Bankov I., Trudgett A. (2011). Genetic diversity of liver flukes (*Fasciola hepatica*) from Eastern Europe. Infect. Genet. Evol..

[b0170] Thurston, M.I., Field, D., 2005. Msatfinder: detection and characterisation of microsatellites. Distributed by the authors at <http://www.genomics.ceh.ac.uk/msatfinder/>. CEH Oxford, Mansfield Road, Oxford OX1 3SR.

[b0175] Valentim C.L., LoVerde P.T., Anderson T.J., Criscione C.D. (2009). Efficient genotyping of *Schistosoma mansoni* miracidia following whole genome amplification. Mol. Biochem. Parasitol..

[b0180] van Dijk J., Sargison N.D., Kenyon F., Skuce P.J. (2010). Climate change and infectious disease: helminthological challenges to farmed ruminants in temperate regions. Animal.

[b0185] van Oosterhout C., Hutchinson W.F., Wills D.P.M., Shipley P. (2004). Micro-checker: software for identifying and correcting genotyping errors in microsatellite data. Mol. Ecol. Notes.

[b0190] Vilas R., Vazquez-Prieto S., Paniagua E. (2012). Contrasting patterns of population genetic structure of *Fasciola hepatica* from cattle and sheep: implications for the evolution of anthelmintic resistance. Infect. Genet. Evol..

[b0195] Walker S.M., Johnston C., Hoey E.M., Fairweather I., Borgsteede F., Gaasenbeek C., Prodöhl P.A., Trudgett A. (2011). Population dynamics of the liver fluke, *Fasciola hepatica*: the effect of time and spatial separation on the genetic diversity of fluke populations in the Netherlands. Parasitology.

[b9005] Weir B.S., Cockerham C.C. (1984). Estimating F-statistics for the analysis of population structure. Evolution.

[b0200] WHO, 2012. Accelerating Work to Overcome the Global Impact of Neglected Tropical Diseases; A Roadmap for Implementation – Executive Summary.

[b0205] WHO, 2007. Action Against Worms.

[b0210] WHO, 2006. Report of the WHO Informal Meeting on Use of Triclabendazole in Fascioliasis Control.

[b0215] Xiao N., Remais J., Brindley P.J., Qiu D., Spear R., Lei Y., Blair D. (2011). Polymorphic microsatellites in the human bloodfluke, *Schistosoma japonicum*, identified using a genomic resource. Parasit. Vectors.

