# Supplementary figures and images for: Characterisation of a novel panel of polymorphic microsatellite loci for the liver fluke, Fasciola hepatica, using a next generation sequencing approach
Source: Infect Genet Evol. 2015 Jun;32:298–304. doi: 10.1016/j.meegid.2015.03.014 (PMC4424948; doi:10.1016/j.meegid.2015.03.014)

## Slide 1
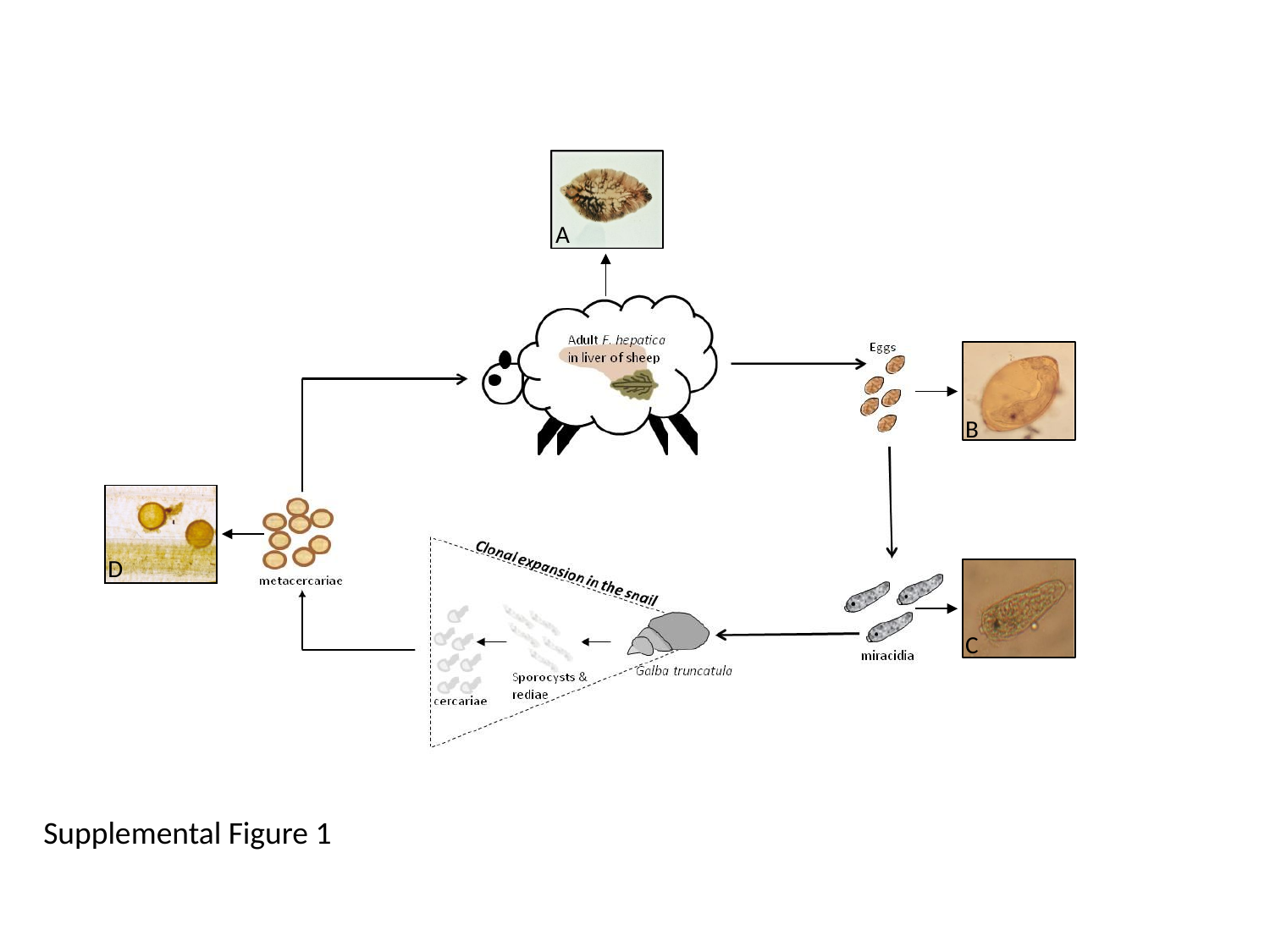

A
B
D
C
Supplemental Figure 1

## Slide 2
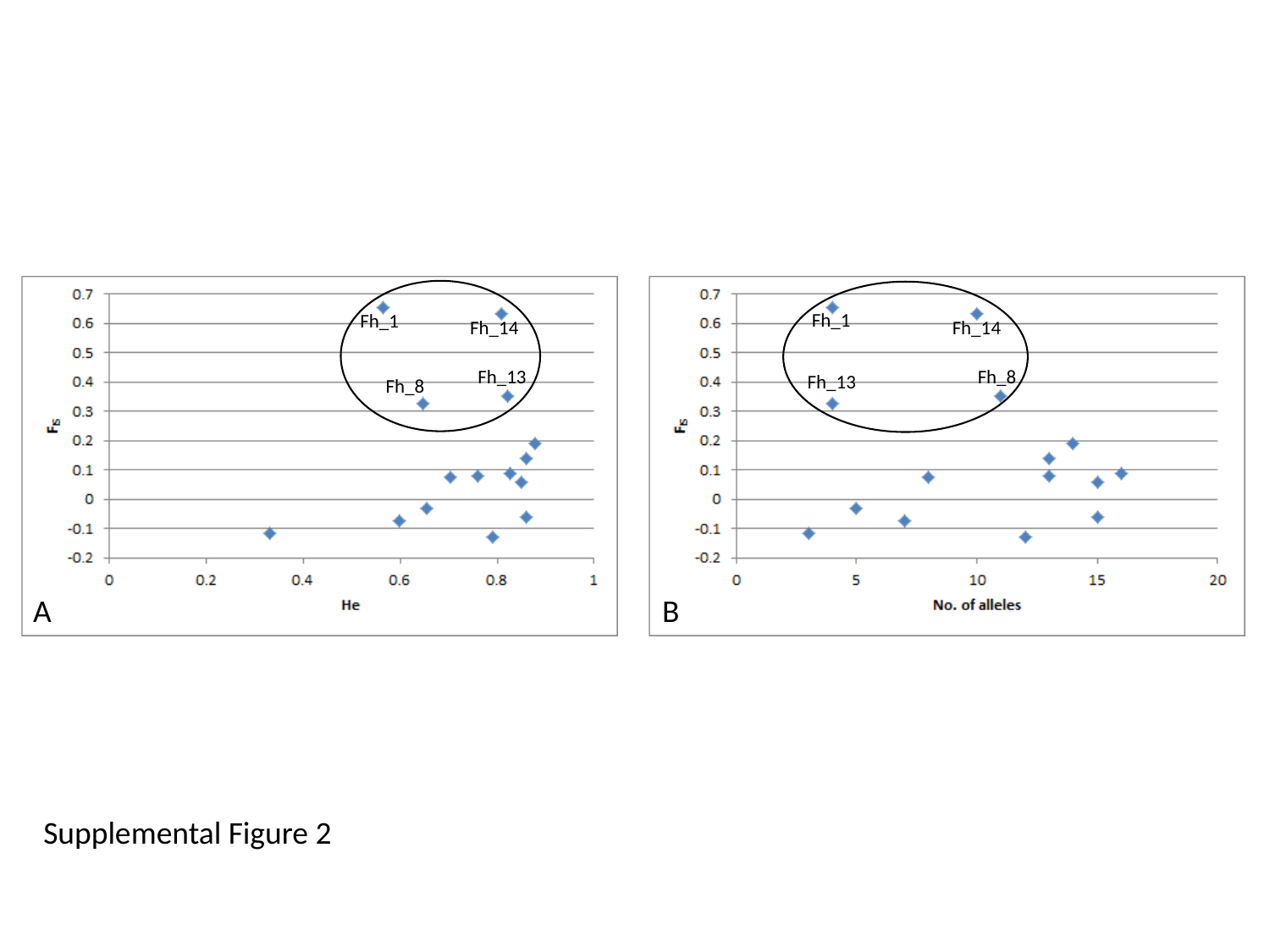

Fh_1
Fh_1
Fh_14
Fh_14
Fh_13
Fh_8
Fh_13
Fh_8
A
B
Supplemental Figure 2

Supplement: Supplementary Figs S1 and S2 [file mmc1.pptx]
