# Supplementary material for: Characterisation of a novel panel of polymorphic microsatellite loci for the liver fluke, Fasciola hepatica, using a next generation sequencing approach
Source: Infect Genet Evol. 2015 Jun;32:298–304. doi: 10.1016/j.meegid.2015.03.014 (PMC4424948; doi:10.1016/j.meegid.2015.03.014)
Supplement: Supplementary Table 1 [file mmc2.docx]

Supplementary Table 1: Representation of the types and number of microsatellite loci identified in the *F. hepatica* genomic sequence.

| Motif type | Threshold settings for number of repeats | Numbers found in genomic sequence |
| --- | --- | --- |
| Di nucleotide repeat | 8 | 830 |
| Tri-nucleotide repeat | 5 | 1346 |
| Tetra-nucleotide repeat | 5 | 194 |
| Penta-nucleotide repeat | 5 | 61 |
| Hexa-nucleotide repeat | 5 | 17 |
| Total |  | 2448 |

Supplementary Table 2: *F. hepatica* (Fh) microsatellites; a multiplex approach

|  |  | Forward primer (5’-3’) | Reverse primer (5’-3’) | T_a_ (°C) |
| --- | --- | --- | --- | --- |
| A | Fh_1 | VIC-CCCATGGTGTTGCACAGAT | CTTCACCAAAAGCCGCTAAC | 55 |
|  | Fh_6 | 6FAM-ACGTCCGTCCGTTAAGTGAG | TTTGAGGTCGACATCCTTCA |  |
|  | Fh_13 | PET-GAAACTGTCCCGAAAACGAG | GCGTGCAACATAGGTGAAAA |  |
|  | Fh_15 | NED-AATGCGGAAAAGAGCGATTA | GAAATTGGGAGCAACTGCAT |  |
| B | Fh_2 | VIC-TGAGAAACTGATTCACCGACTG | GAGCTTGTGCTCTCGGAACTA | 57 |
|  | Fh_3 | PET-CACGGCAACTGATGAATGAA | TCCTCGTTTTTGGACCTCAG |  |
|  | Fh_5 | 6FAM-CATCACCACTGTCTTCGATCA | CGAAGCATTGATAAGATTTCCA |  |
|  | Fh_8 | NED-CTCCTGAGGATGATCGGAAA | CTACCGGATCGTTTTGACCA |  |
| C | Fh_9 | PET-AACCCGTATCACCACCAAAC | CTCCCAATCCTGCCATACAT | 57 |
|  | Fh_10 | NED-TTTAGTCGCGGAGCTACCAT | CCACTTTCGTCATGCACATT |  |
|  | Fh_11 | 6FAM-TAAACCGTTGCTTCACGTTG | CAAAGTGTTTGGCGAGCTG |  |
|  | Fh_14 | VIC-ACAGGGCTTTGAAGCATGAC | GGGATAGAGCCGTACTGGAA |  |
| D | Fh_4 | PET-TCACGAAATTGGAGACGACA | TGCATGCAAGAATTGTACCC | 59 |
|  | Fh_7 | NED-TGCACTCTAGCATGGTTTGG | AAGTCTTCAGTGCCCCTTCC |  |
|  | Fh_12 | VIC-CCACGAGAAGTGGAATTCGT | GTAGGTCCACTCCCTGTCCA |  |

T_a_: Annealing temperature for PCR.
